# Education and health of children with hearing loss: the necessity of signed languages

**DOI:** 10.2471/BLT.19.229427

**Published:** 2019-08-20

**Authors:** Joseph J Murray, Wyatte C Hall, Kristin Snoddon

**Affiliations:** a Department of American Sign Language and Deaf Studies, Gallaudet University, 800 Florida Ave NE, Washington, DC, 20002, United States of America (USA).; b Department of Obstetrics and Gynecology, University of Rochester Medical Center, Rochester, USA.; c School of Early Childhood Studies, Ryerson University, Toronto, Canada.

## Abstract

Medical and educational interventions for children with hearing loss often adopt a single approach of spoken language acquisition through the use of technology, such as cochlear implants. These approaches generally ignore signed languages, despite no guarantees that the child will acquire fluency in a spoken language. Research with children who have a cochlear implant and do not use a signed language indicates that language outcomes are very variable and generally worse than their non-deaf peers. In contrast, signing children with cochlear implants have timely language development similar to their non-deaf peers that also exceeds their non-signing peers with cochlear implants. Natural signed languages have been shown to have the same neurocognitive benefits as natural spoken language while being fully accessible to deaf children. However, it is estimated less than 2% of the 34 million deaf children worldwide receive early childhood exposure to a signed language. Most deaf children are, therefore, at risk for language deprivation during the critical period of language acquisition in the first five years of life. Language deprivation has negative consequences for developmental domains, which rely on timely language acquisition. Beyond the adverse effects on a child’s education, language deprivation also affects deaf people’s mental and physical health and access to health care, among others. Therefore, policies in accordance with the United Nations *Convention on the rights of persons with disabilities* are needed. Such policies would ensure early intervention and education services include signed languages and bilingual programmes where the signed language is the language of instruction.

## Résumé

Les interventions médicales et éducatives destinées aux enfants souffrant d'une perte de l'acuité auditive s'inscrivent souvent dans une approche unique d'acquisition du langage parlé par des moyens technologiques, comme les implants cochléaires. Ces approches font généralement abstraction des langues des signes alors que rien ne garantit que l'enfant parvienne à maîtriser le langage parlé. Les recherches menées auprès d'enfants qui ont reçu un implant cochléaire et n'utilisent pas de langue des signes montrent que les résultats langagiers sont très variables et généralement pires que ceux des enfants entendants. En revanche, les enfants qui ont reçu un implant cochléaire et utilisent des signes ont un développement langagier en temps voulu similaire à celui des enfants entendants et supérieur à celui des enfants dotés d'un implant cochléaire qui n'utilisent pas de signes. Il a été démontré que les langues des signes naturelles procurent les mêmes bienfaits neurocognitifs que la langue parlée naturelle tout en étant pleinement accessibles aux enfants sourds. On estime néanmoins que moins de 2% des 34 millions d'enfants sourds à travers le monde sont exposés à une langue des signes au cours de leur petite enfance. La plupart des enfants sourds risquent donc de souffrir d'une carence langagière lors de la période critique de l'acquisition du langage, au cours des cinq premières années de vie. La carence langagière a des conséquences négatives sur les domaines du développement qui dépendent d'une acquisition du langage en temps voulu. Au-delà des effets négatifs sur l'éducation de l'enfant, la carence langagière a aussi une incidence sur la santé mentale et physique des sourds et sur leur accès aux soins de santé, notamment. Par conséquent, des politiques conformes à la Convention des Nations Unies relative aux droits des personnes handicapées sont nécessaires. Ces politiques garantiraient l'inclusion par les services d'intervention précoce et d'éducation d'une langue des signes et de programmes bilingues dans le cadre desquels la langue des signes serait la langue d'enseignement.

## Resumen

Las intervenciones médicas y educativas para niños con pérdida auditiva suelen adoptar un único enfoque de adquisición del lenguaje hablado mediante el uso de tecnología, como los implantes cocleares. Estos enfoques generalmente ignoran los lenguajes de señas, a pesar de que no hay garantías de que el niño adquiera fluidez en un lenguaje hablado. La investigación en niños que tienen un implante coclear y no usan lenguaje de señas indica que los resultados del lenguaje son muy variables y generalmente peores que los de sus compañeros sin problemas de audición. Por el contrario, los niños que usan el lenguaje de señas y tienen implantes cocleares se pueden desarrollar a tiempo de manera similar a sus compañeros sin problemas de audición, y también superan a sus compañeros con implantes cocleares que no usan el lenguaje de señas. Se ha demostrado que los lenguajes naturales de señas tienen los mismos beneficios neurocognitivos que el lenguaje natural hablado, a la vez que son totalmente accesibles para los niños sordos. Sin embargo, se estima que menos del 2 % de los 34 millones de niños sordos en todo el mundo están expuestos a un lenguaje de señas en la primera infancia. Por lo tanto, la mayoría de los niños sordos corren el riesgo de sufrir privaciones del lenguaje durante el periodo crítico de adquisición del lenguaje en los primeros cinco años de vida. La privación del lenguaje tiene consecuencias negativas para los dominios del desarrollo que dependen de la adquisición oportuna del lenguaje. Más allá de los efectos adversos sobre la educación de un niño, la privación del lenguaje también afecta a la salud mental y física de las personas sordas y al acceso a la atención sanitaria, entre otras cosas. Por consiguiente, se necesitan políticas acordes con la Convención de las Naciones Unidas sobre los derechos de las personas con discapacidad. Estas políticas garantizarían que los servicios de intervención temprana y educación incluyan el lenguaje de señas y programas bilingües en los que el lenguaje de señas sea el idioma de instrucción.

## ملخص

غالبًا ما تنتهج التدخلات الطبية والتعليمية للأطفال الذين يعانون من فقدان السمع طريقة وحيدة لاكتساب اللغة المنطوقة الاستحواذ من خلال استخدام التكنولوجيا، مثل حالات زراعة القوقعة. تتجاهل هذه الأساليب بشكل عام اللغات الموقعة، على الرغم من عدم وجود ضمانات بأن الطفل سوف يكتسب الطلاقة في اللغة المنطوقة. إن الأبحاث على الأطفال الذين لديهم غرس للقوقعة ولا يستخدمون لغة موقعة، تشير إلى أن نتائج اللغة متغيرة للغاية وأسوأ بشكل عام عن مثيلاتها في أقرانهم الذين لا يعانون من الصمم. وعلى طرف النقيض، فإن توقيع الأطفال من ذوي غرس القوقعة لديهم تطور لغوي في الوقت المناسب مشابه لإقرانهم الذين لا يعانون من الصمم، وهو يتجاوز أيضاً أقرانهم غير الموقّعين بغرس قوقعة للأذن. أظهرت اللغات الموقعة الطبيعية أن لديها مزايا الإدراك العصبي مثل اللغة المنطوقة الطبيعية كما أنها تكون في متناول الأطفال الصم تماماً. ومع ذلك، فإنه من المقدر أن أقل من 2٪ من الأطفال الصم البالغ عددهم 34 مليونًا في جميع أنحاء العالم، يتعرضون في مراحل الطفولة المبكرة للغة موقعة. وعلى ذلك فإن معظم الأطفال المصابين بالصمم، يتعرضون لخطر الحرمان من اللغة خلال الفترة الحرجة من اكتساب اللغة في السنوات الخمس الأولى من العمر. يكون للحرمان من اللغة تبعات سلبية على مجالات التطور التي تعتمد على اكتساب اللغة في الوقت المناسب. وبخلاف التأثيرات السلبية على تعليم الطفل، فإن الحرمان من اللغة يؤثر أيضًا على الصحة العقلية والبدنية للأشخاص المصابين بالصمم، وحصولهم على الرعاية الصحية، بالإضافة إلى مميزات أخرى. ولذلك، فإن هناك حاجة إلى السياسات التي تتوافق مع اتفاقية الأمم المتحدة لحقوق الأشخاص من ذوي الإعاقة. سوف تضمن هذه السياسات أن التدخل المبكر والخدمات التعليمية تشمل لغة موقعة وبرامج ثنائية اللغة حيث تكون اللغة الموقعة هي لغة التدريس.

## 摘要

针对失聪儿童的医疗和教育干预通常是使用人工耳蜗等技术、采用单一的口头语言习得方法。通常情况下，这些方法忽略了手语，尽管手语的习得也无法保证孩子能够流利地使用口语。一项对植入人工耳蜗，但没有使用手语的儿童进行的研究表明，语言习得的结果有很大差异，一般要比他们没有失聪的同龄人更差。相比之下，对植入人工耳蜗，同时也使用手语的儿童来说，他们的及时语言习得与没有失聪的同龄人相当，也超过了植入人工耳蜗但没有使用手语的同龄失聪儿童。经验证，自然手语显现出与自然口语相同的神经认知优势，而且失聪儿童完全能够使用。据估计，在全球 3400 万失聪儿童中，只有不到 2% 的儿童在幼儿时期接触过手语。因此，大多数失聪儿童在语言习得的关键时期（出生后的五年）面临着语言剥夺的风险。语言剥夺对依赖于及时语言习得的发展性领域有负面影响。除了对儿童教育有不利影响之外，语言剥夺还会影响失聪者的身心健康以及获得医疗保健的机会等。因此，我们需要根据联合国《残疾人权利公约》制定相应政策。此类政策将能确保包括手语和双语课程（其中手语是授课语言）在内的早期干预和教育服务。

## Резюме

Медицинские и образовательные мероприятия для детей с потерей слуха часто предполагают единственный подход — получение возможности воспринимать устную речь с помощью технологических решений, например кохлеарных имплантов. Такие подходы обычно игнорируют использование языка жестов, несмотря на отсутствие гарантий того, что ребенок получит возможность свободно говорить. Исследования с участием детей с кохлеарными имплантами, не использовавших язык жестов, показали, что результаты развития речи очень изменчивы и в целом хуже, чем у сверстников, не страдающих потерей слуха. Напротив, дети с кохлеарными имплантами, владеющие языком жестов, характеризовались своевременным развитием речи, сходным с таковым для сверстников без потери слуха и превышавшим таковое у сверстников с кохлеарными имплантами, но не использовавшими язык жестов. Показано, что естественные языки жестов обладают теми же нейрокогнитивными преимуществами, что и естественная устная речь, будучи в то же время полностью доступными для детей с потерей слуха. Следует отметить, что менее 2% из 34 миллионов детей с потерей слуха во всем мире получают в раннем детстве доступ к языку жестов. Таким образом, большинство детей с потерей слуха подвергаются риску речевой депривации в течение критического периода освоения языка в первые пять лет жизни. Речевая депривация характеризуется негативными последствиями для аспектов развития, связанных со своевременным освоением языка. Кроме негативного влияния на образование ребенка, речевая депривация также нарушает умственное и физическое здоровье людей с потерей слуха и среди прочего доступ к средствам здравоохранения. Таким образом, необходимы правила, соответствующие Конвенции Организации Объединенных Наций о правах инвалидов. Такие правила могли бы обеспечить раннее вмешательство и включение языка жестов в образовательные услуги, а также создание билингвальных программ, где язык жестов использовался бы в качестве языка инструктирования.

## Introduction

When infants are identified with hearing loss, the primary concern is often their language development and the developmental domains that rely on timely language acquisition, such as cognition and socioemotional development. This concern can quickly become a developmental emergency if deaf children are unable to access the spoken language within their home and their non-deaf family members do not know a signed language. Typical medical and educational interventions are to address the hearing loss via technology, such as hearing aids and cochlear implants, often without incorporating the learning of a signed language. In this article, we argue that relying solely on the use of spoken language with hearing loss technology creates conditions that elevate the risk of poor language acquisition. We discuss language acquisition as a human rights issue for deaf people and urge policy-makers to ensure deaf children gain better access to natural signed languages to promote their healthy development.

## Signed languages

Signed languages are natural human languages existing across numerous societies around the world. As with spoken languages, signed languages display phonetic, phonemic, syllabic, morphological, syntactic, discourse, and pragmatic levels of organization as expected of natural languages.^1^ While often named using national terms (such as American Sign Language, Thai Sign Language, among many others), signed languages are distinct from spoken languages and there can be several signed languages in one country. Several hundred signed languages are estimated to exist today. These visuospatial languages have helped scholars understand languages as shared human phenomena, which can emerge in both spoken and visual forms. Signed languages are widely used by people, and have emerged throughout human history as shared languages in communities of deaf and non-deaf users.^2^ In the present day, widespread interest in signed languages has led to a large and growing number of non-deaf people becoming signed language users in many countries around the world.^3^

For what are often ideological reasons, policies on early intervention and education typically fail to support signed language acquisition in deaf and hard-of-hearing children.^4^^,^^5^ Early, immersive exposure to a natural language is important for timely neurocognitive and linguistic development of any child. For most deaf children, access to a signed language is an important precondition for this development since unhindered access to a spoken language is not possible.^6^^,^^7^ Data tracking of the global deaf population is minimal and a lack of reliable data is part of the problem. Currently, best estimates, from a survey of 37 828 students, suggest that less than 6% of deaf children in the United States of America (USA) receive access to a signed language in early childhood.^8^ Global reports also highlight a stark picture, with an estimated less than 2% of 34 million deaf children worldwide receiving access to a signed language in early childhood.^9^ In sum, there is likely a high proportion of deaf children globally at risk of experiencing language deprivation.

## Language deprivation

Language deprivation is the persistent lack of unhindered access to a natural language during the critical period of language acquisition. This period of approximately the first five years of life is a time-limited window of brain development for establishing first-language fluency.^10^ The research evidence strongly suggests that language deprivation, rather than strictly hearing loss, is the underlying cause of poor language, educational and health outcomes in the deaf population, and is traceable to a lack of a signed language exposure for deaf children in their early development.^11^ Additionally, concerns related specifically to developmental delays, attention-deficit disorder and socioemotional difficulties continue to dominate the development of deaf children.^12^^,^^13^ Finally, permanent neurostructural differences (such as less myelination of neurolinguistic pathways) also exist in deaf people who do not acquire fluency in a spoken language and who experience delayed exposure to a signed language when compared with those who have timely acquisition of signing.^14^

Overall, the current evidence suggests that auditory and oral-only approaches, which reject early, immersive use of signed languages do not promote ideal developmental outcomes in deaf children. Furthermore, when these efforts are unsuccessful, the effects of language deprivation cannot be entirely redressed by later use of a signed language as a backup plan.^15^ Instead, signed languages appear to be important not only for optimal development of deaf children, but also for achieving the desired outcomes of cochlear implants, as signing children with cochlear implants outperform non-signing children with implants in speech and language outcomes.^16^

Since the late 1980s, cochlear implants have quickly become the standard of care in high-income countries, and the technology is now rapidly being exported to low- and middle-income countries.^17^ There is frequently ideological resistance to the use, and lack of understanding, of signed languages among medical and education professionals who promote spoken language-only approaches, and the use of cochlear implants. This mind-set is likely to influence parental decision-making regarding deaf children’s learning of signed languages.^4^^,^^5^ These professional views are held despite evidence of highly variable, delayed spoken language outcomes in non-signing children with cochlear implants and of good spoken language outcomes in children with cochlear implants who are exposed to a signed language from birth.^11^^,^^16^^,^^17^

The global community’s failure to support the acquisition of signed languages by deaf and hard-of-hearing children has adverse, lifelong effects on education, socioemotional, and cognitive development.^15^ The unpredictable outcomes of cochlear implants in supporting language development of non-signing children suggest that these adverse effects are not prevented, fully remediated or cured by hearing-loss technology, such as cochlear implants.^6^^,^^11^^,^^16^^–^^18^ Parents are then left to make decisions about learning either a signed or spoken language “without any guarantees about the level of benefit their children will receive from having [cochlear implants].”^19^

## Cognitive development

First-language acquisition in deaf children via an accessible signed language is often a precondition for their ability to understand and use spoken and written languages.^10^ Generally, deaf children with optimal exposure to a signed language will achieve expected development milestones.^11^ In fact, signing children with implants demonstrate better speech and language development than non-signing children with implants and similar development as non-deaf children.^16^^,^^17^ In the case of absent or incidental language exposure, including to a signed language, there is a greater likelihood of delays in cognitive development. These problems include delays in social cognition skills such as Theory of Mind abilities, which allow a child to recognize different mental states and perceptions in others, and general delays in academic learning and achievement.^20^ Individuals who experience language deprivation syndrome may have language dysfluency, general knowledge gaps, disruptions in thinking, mood or behaviour, and general delays in development of academic or literacy skills. These symptoms and others demonstrate the absence of typical cognitive structures and disordered behaviours in children who do not have unhindered early developmental language exposure.^10^^,^^21^

## Socioemotional development

For deaf children, accessible communication with their parents and peers supports their social and emotional development, including enhanced self-esteem and ability to build relationships.^22^ Research demonstrating a greater prevalence of mental health problems in deaf populations also underlines the importance of early effective communication with family members and peers.^7^ Chronic lack of accessible communication with non-deaf parents is commonly reported as a childhood emotional trauma by deaf adults.^23^ Generally deaf children in non-signing families are vulnerable to the “dinner table syndrome,” a label that describes the chronic experience of observing spoken conversations between other family members and not understanding what is said. A retrospective study of 211 American deaf adults found having non-deaf parents increases the likelihood of reported experiences of dinner table syndrome by 17.6-fold. Furthermore, whether or not children had cochlear implants or hearing aids had no influence on this result.^12^ Due largely to a lack of comprehensive support for signed language learning, non-deaf parents in the USA are three times less likely to use a signed language with their deaf child than do deaf parents.^24^

Lack of language access in early childhood places deaf children and adults at risk of further adverse health effects. Generally, deaf individuals are at greater risk of experiencing emotional and physical neglect and abuse, sexual trauma and higher rates of depression and anxiety.^25^ As well as a general risk of poorer overall mental health,^7^ deaf people are at greater risk of obesity and increased use of hospital emergency departments, among other problems.^26^ Health-system barriers that affect many minority-language populations also affect this population. Such barriers include poor access to primary care; lack of qualified interpreters; doctors using inaccessible medical terminology; and written after-visit paperwork. These barriers, however, are not only exacerbated by barriers to services in signed languages, but are also compounded by the cognitive and social effects of language deprivation.

## Access to bilingual education

There are few data, even in high-income countries, on deaf children’s access to bilingual educational settings where a sign language is the language of instruction. The World Health Organization reports that children with sensory disabilities face higher barriers to education than those with physical disabilities and, thus subsequently, have lower enrolment rates in schools.^27^ Of the 34 million children with disabling hearing loss around the world,^28^ the World Federation of the Deaf estimates 80% do not have access to education. Of the 20% who do have access, very few are educated in bilingual programmes that incorporate their national signed and spoken or written languages as languages of instruction and study.^9^ In Burkina Faso, 90% of deaf 7- to 12-year olds were not in school in 2006. In Rwanda, only 309 deaf children out of an estimated 10 000 in the country were reported to be attending school in 2007.^28^^,^^29^

The 2006 United Nations *Convention on the rights of persons with disabilities*^30^ obliges governments to secure the human right to a signed language in legislation; to ensure its use in civil society, educational settings and cultural settings; and to secure its use as a means of access to wider society, including health services. To ensure deaf children’s access to their rights, there exists a specific requirement in the Convention that they should be educated in languages that are fully accessible to them and in environments that maximize their physical, cognitive, academic and social development. Such educational settings are required to promote deaf children’s linguistic identity and facilitate their learning of a signed language with deaf teachers who are themselves fluent signers.^31^

## Promoting signed language access

Optimal outcomes in policy-making for deaf signers should follow international standard best practices. Article 21 of the *Convention on the rights of persons with disabilities*

calls upon governments to promote the use of signed languages, as an integral part of the right to freedom of expression and to seek, receive and impart information. Article 30, regarding health, also calls on governments to provide early intervention services designed to minimize and prevent further disabilities. Best practices in health-care access should follow these Articles, as well as Article 9, which requires the provision of accessibility in health-care settings via national signed language interpreters. The removal of structural and socioeconomic barriers to learning a signed language in early childhood are essential to ensuring access to education for children with hearing loss. Equally important are the provisions of Article 24, outlining the need to ensure children have language-rich settings with signed language-using peers and teachers who can provide direct access to the curriculum in sign languages.^29^ Current research demonstrates that many deaf children raised without a signed language cannot achieve age-appropriate linguistic fluency, even with high-quality spoken language interventions.^11^

Early and consistent exposure to signed languages provides deaf children with fully accessible language exposure, avoiding the creation of an artificial ceiling on developmental and educational outcomes that are too often simply blamed on hearing loss. Access to a signed language enhances the overall effectiveness of global screening and intervention systems, as well as fully inclusive education and health care for deaf children and adults. Policy-makers should ensure that deaf children and their families are given unhindered access to signed languages as a primary option in all early intervention measures. This access should continue throughout the child’s development and educational career, and across the lifespan.
